# Unveiling a novel parasitosis: *Trichostrongylus colubriformis* infection in captive ring-tailed lemurs (*Lemur catta*)

**DOI:** 10.1016/j.ijppaw.2023.11.003

**Published:** 2023-11-28

**Authors:** Filippo Maria Dini, Monica Caffara, Matteo Galliani, Chiara Cotignoli, Michele Capasso, Perla Tedesco, Roberta Galuppi

**Affiliations:** a Department of Veterinary Medical Sciences (DIMEVET), Alma Mater Studiorum University of Bologna, Via Tolara di Sopra 50, 40064 Ozzano Emilia (BO), Italy; b DVM, Safari Ravenna, Via dei Tre Lati 2X, 48125 Ravenna, Italy

**Keywords:** Lemur parasites, *Trichostrongylus colubriformis*, Zoo animals, Parasite transmission

## Abstract

Ring-tailed Lemur (*Lemur catta*) is the only surviving semi-terrestrial diurnal lemur in Madagascar. Despite being the most intensively studied of lemur taxa, only a few helminths have been described in this species. In this study we describe a persistent infection due to *Trichostrongylus colubriformis* in a captive population of *L. catta* hosted in a zoological park of northern Italy. In the context of a parasitological survey on zoo animals, we investigated parasites in a captive colony of ring-tailed lemurs within a zoological park. Parasitological analysis included necropsy of a deceased lemur in 2019, subsequent fecal sample collections in 2021–2022, followed by coprological examination and coprocolture. Morphological and molecular analyses were conducted on adult helminths, larvae and eggs, involving microscopy, scanning electron microscopy (SEM), and sequencing of the ITS rDNA region. Trichostrongylidae parasites were primarily found after necropsy in the intestine of the lemur. Morphological and molecular investigations on adults and eggs/larvae recovered from feces collected at different times from lemurs of the same captive population, allowed to properly identify the parasite as *T*. *colubriformis*. To the best of our knowledge this is the first description of *T. colubriformis* in *L. catta*. Although its presence in wild populations is not necessarily implied by our finding, this parasitosis represent a cause of concern in captive lemurs, considering the possibility of interspecies transmission and the zoonotic implications.

## Highlights

• First report of *Trichostrongylus colubriformis* in captive ring-tailed lemurs.
• Persistent, localized parasite cycle was observed within the lemur population.
• Interspecies transmission is a potential risk in zoo environments.
• Importance of parasite identification for animal health and zoonotic risk assessment.

## Graphical abstract

**Figure:**
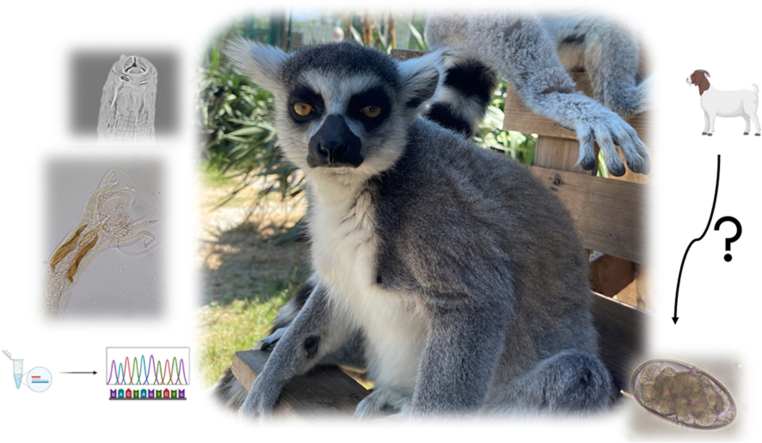

## 1 Introduction

Lemurs (Lemuriformes: Lemuroidea) are a group of enigmatic non-human primate (NHP) endemic to Madagascar where, probably following the arrival of a single common ancestor to the island approximately in Late Cretaceous–Middle Eocene (70–41 million years ago), they have evolved and diversified into several species, some of which are now extinct (Poux et al., 2005). Nowadays in Madagascar five families are known as living, including 15 genera, 99 species, and 103 taxa (including subspecies), distributed into an area of about 50,000–60,000 km^2^ (Schwitzer et al., 2016).

Lemurs have aroused much curiosity among scientists for their nocturnal behaviour and habits, in fact the name *Lemur*, used by Linnaeus to originally describe the species of this group (*Lemur tardigradus*, now in the genus *Loris*), was inspired by the Latin term *lemures*, ghost or spectre of the night of the Roman mythology (Dunkel et al., 2011/12).

The Ring-tailed Lemur (*Lemur catta* Linnaeus, 1758) of the family Lemuridae, is the most known species, characterized by a diurnal activity, ability to climb, and considered the most terrestrial among lemurs. Despite their large range and ecological flexibility, which allows the colonization of diverse habitats in southern, south-west, and south-central Madagascar, population density is low and restricted to isolated fragments throughout the geographical range (Davies and Schwitzer, 2013). This species has recently been assessed for the IUCN Red List of Threatened Species (2018) and listed as Endangered under criteria A4cd (LaFleur and Gould. 2020). The entire family Lemuridae (large lemurs) are listed on CITES 1 appendix.

*Lemur catta* is easily bred and raised in captivity, it is present in zoos worldwide (http://www.isis.org), and could serve as a source of lemurs to be reintroduced in the wild if necessary (Taylor, 2009). An experimental release program took place in 1985, when the Wildlife Conservation Society introduced ring-tailed lemurs from the Bronx Zoo in the St. Catherine's Island, Georgia, USA, revealing the ability of this species to readily adapt to their new environment and to exhibit the broad repertoire of behaviors seen in naïve populations (Keith-Lucas et al., 1999). However, the hypothesis regarding the introduction or reintroduction of animals into their natural habitat often tends to underestimate the influence of parasitic fauna originating from captive individuals on the wild population (Villers et al., 2008).

Despite the extensive research carried out on the ecology and phylogenesis of lemurs in Madagascar, the knowledge on their parasitic fauna is still limited. Most studies on helminth parasites of lemurs dated back to 1950s and 1960s (Chabaud and Petter, 1958, 1959; Chabaud et al., 1961a, 1961b, 1964, 1965) and were aimed to describe the presence of parasites by examining adult worms from necropsies performed on either deceased captive animals, or on individuals hunted, with permission, specifically for this purpose (Irwin and Raharison, 2009). In their review on the endoparasites of Madagascar lemur species, the latter authors reported 27 helminths and 12 protozoans collected from different wild lemur species. To adhere to ethical restrictions, recent research has focused on non-invasive sampling techniques such as fecal analysis, nevertheless these studies often lack specific identification of the parasites found (Villers et al., 2008; Loudon and Sauther, 2013).

Despite being the most intensively studied of lemur taxa, only few helminths have been described in *L. catta*: Chabaud et al. (1965) reported only the presence of *Trichuris lemura*; in Loudon et al. 2006 during a survey carried out on wild ring-tailed lemur, described the presence of pinworm and strongylid eggs, tentatively identified as *Lemuricola* sp. and *Lemurostrongylus* sp. respectively, and coccidia oocysts; in Robles et al., 2010 found the oxyurid *Lemuricola bauchoti*, and Loudon and Sauther (2013) reported unidentified trichostrongylid eggs and *Subulura* sp. Villers et al. (2008), observed a significant difference in gastrointestinal parasites between wild and captive populations of *L. catta* through fecal examination. In fact, strongyle-type eggs were found exclusively in captive lemurs, while pinworm-type eggs were present only in wild lemurs. However, no further identification of the specific parasites was reported.

The possibility that captive lemurs, like other non-human primate species, may host non-specific parasites should be taken into consideration; this could be related to inadequate isolation of other captive animal species or to the access of wild animals to lemur enclosures. Moreover, the ingestion of contaminated food, such as fresh vegetables or water, containing infective stages of various parasites cannot be ruled out. In Italy, larval forms of *Taenia martes* (De Liberato et al., 2014) and *Echinococcus granulosus* (Poglayen et al., 2016) were detected during necropsies of *L. catta* from two different zoological parks. These findings were linked to the ingestion of food contaminated with eggs from wild animals, highlighting the potential transmission of parasites through food sources.

In the present study we describe the presence of persistent infection by *Trichostrongylus colubriformis* in a population of captive lemurs hosted in a natural park in Northern-Italy.

## 2 Materials and methods

### 2.1 Source material and sampling area

In the framework of a parasitological survey on zoo animals, we had the chance to study a captive colony of ring-tailed lemurs (*Lemur catta*) hosted in the Zoo Safari premises (Ravenna province, Northern-eastern Italy: 44°19′35.5''N; 12°16′29.9''E). The lemurs were housed in an area called “Lemur Island,” composed of 30 subject, part of the pedestrian zone where a population of approximately 15–20 free-ranging goats is also present. The lemurs are kept in a walk-through enclosure covered by nets, with vegetation, wooden shelter as refuge, and environmental enrichment items; they are fed with vegetables and fruits grown within the area, along with tap water.

In June 2019, a necropsy was performed on a naturally deceased three months old ring-tailed lemur. The intestine was examined to investigate the presence of parasites by opening lengthwise, and scraping the mucosa; the content was then examined under a dissecting microscope. The helminths collected from the gut, were observed under a dissection microscope to evaluate the gross morphology, then under a light microscope (Leica Microsystems, Wetzlar, Germany) to record total length (TL) with the aid of a digital Nikon DS-Fi1 camera and image-acquisition software (Nikon Nis-Elements D3.0), also used for further morphometrics. A small part, about 5 mm, from 10 males and 10 females was excised from the central portion of the nematodes, where taxonomical features are not present, for molecular studies. The anterior and posterior portions of the parasites body were clarified in Amman's lactophenol to measure the internal taxonomical features by light microscope. Morphometric analysis was carried out following Travassos (1921). For scanning electron microscopy (SEM), anterior and posterior portion of one nematode were dehydrated through a graded ethanol series, subjected to critical point drying, sputter-coated with gold palladium, and observed using a Phenom XL G2 Desktop SEM (Thermo Fisher Scientific, Eindhoven, The Netherlands) operating at 5 kV (Davidovich et al., 2022).

In June 2021 and July 2022, two pooled samples of lemurs feces were collected from the ground during each sampling event while in November 2022, 10 individual fecal samples were collected directly from the ground during a 1-h observation period. Additionally, two pooled samples of feces from free-ranging goats present in the pedestrian area were also collected in July and November 2022 (Table 1). All fecal samples were collected using plastic bags and refrigerated until examination.

**Table 1:** Description of the sampling events, methods and results obtained in this study.

Sampling date	Animals	Matrices	Flotation +	McMaster	Morphological results	Coproculture	PCR	Sequencing
Dec 2019	Lemur	Intestine	–	–	178 adults (*T. colubriformis*)	–	20 adults	*T. colubriformis* (3)
June 2021	Lemur	Feces (2 pools)	1 pool	–	Trichostrongylid	–		–
July 2022	Lemur	Feces (2 pools)	2 pools	–	Trichostrongylid	Two 3rd stage larvae	eggs + L3	*T. colubriformis*
	Goat	Feces (2 pools)	2 pools	–	Trichostrongylid		eggs	Mixed pherogram
Nov 2022	Lemur	Feces (10 samples)	5 out of 10	<20–200 epg	Trichostrongylid	–	–	–
	Goat	Feces (2 pools)	2 pools	–	Trichostrongylid	One 3rd stage larvae	L3	*O. venulosum*

All the fecal samples underwent the flotation technique and, the individual fecal samples collected in November 2022 immediately after defecation were also subjected to egg quantification using the McMaster technique. Coproculture was performed on fecal pools tested positive for strongylid eggs from both lemurs and goats during the last two sampling events in 2022. To extract the larvae, the Baermann's technique was employed. The third-stage larvae of parasitic nematodes were then separated from the free-living ones using the MAAF manual method (1986). Gastrointestinal strongyle larvae were carefully collected under a dissection microscope and stored at −20 °C for further molecular analyses.

### 2.2 Molecular examination

For molecular analysis, genomic DNA was extracted from 20 central pieces of adult nematodes (10 males and 10 females), from 3 larvae extracted with Baermann technique from coprocultures of lemurs (2) and goats (1), and from some strongylid eggs from lemurs and goats, using a PureLink® Genomic DNA Kit (Life Technologies, Carlsbad, CA, USA) following the manufacturer's instructions.

Amplification of the ITS rDNA region was performed with the primers NC5_f (5′-GTAGGTGAACCTGCGGAAGGATCATT-3′) and NC2_r (5′-TTAGTTTCTTCCTCCGCT-3′) (Zhu et al., 1998). The PCR products were electrophoresed on a 1% agarose gel stained with SYBR Safe DNA Gel Stain (Thermo Fisher Scientific, Carlsbad, CA, USA) in 0.5 × TBE. For sequencing, the amplicons were excised and purified by Nucleo-Spin Gel and PCR Clean-up (Mackerey-Nagel, Düren, Germany), and sequenced with an ABI 3730 DNA analyzer (StarSEQ, Mainz, Germany). The DNA trace files were assembled with Contig Express (VectorNTI Advance 11 software, Invitrogen, Carlsbad, CA, USA), and the consensus sequences were compared with published data by BLAST tools. Multiple sequence alignments were performed using BioEdit 7.2.5, maximum-likelihood (ML) tree (T96 + G substitution model and bootstrap of 1000 replicates) was obtained using MEGA 7.

The sequences generated in this study have been deposited in GenBank under accession numbers OR352129-34.

## 3 Results

**Fig. 1:**
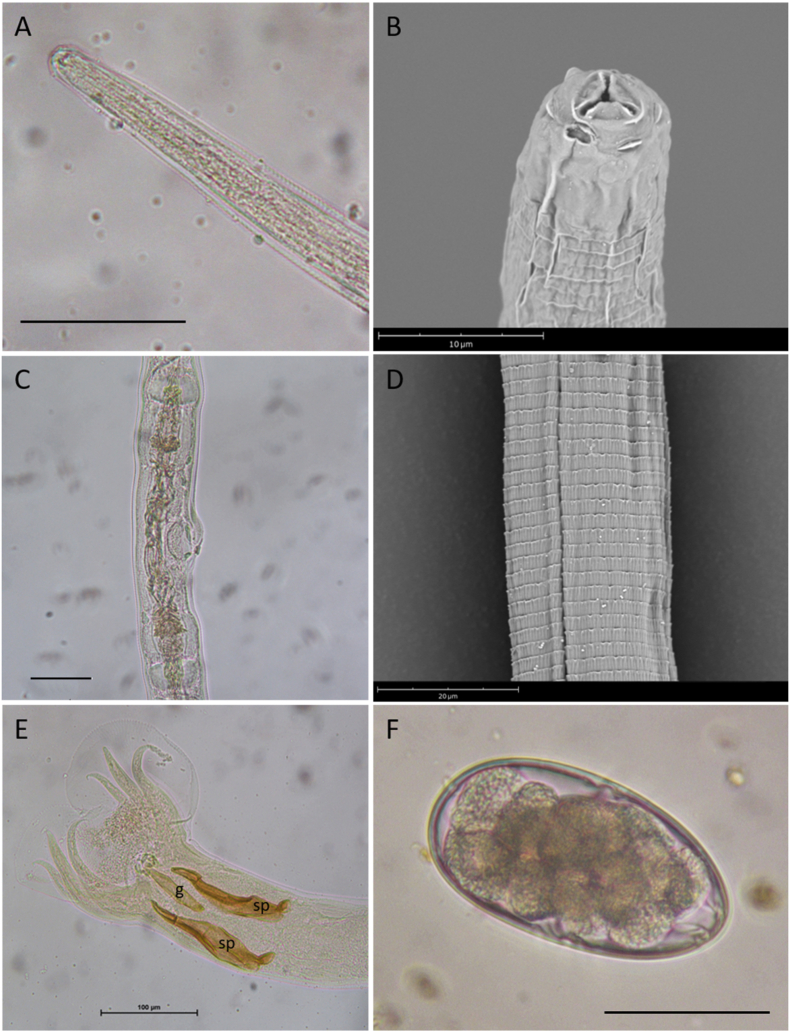
Morphology of *Trichostrongylus colubriformis* adult stages: A) anterior end, scale bar 50 μm; B) detail of anterior end of female showing three small lips and a circle of cephalic papillae; C) detail of ovijector, scale bar 50 μm; D) detail of the cuticle; E) posterior end of male showing the structure of bursa, the gubernaculum (g) and short subequal spicules (sp); F) egg, scale bar 50 μm.

A total of 178 adults (117 females and 61 males) bursate Nematoda were collected from the intestinal contents and based on the morphometric characters they were identified as *Trichostrongylus colubriformis*. The main morphological characters were as follows: females (n = 10) 5559.1 ± 391.2 (4.888–6.236) μm total length; males (n = 10) 4597 ± 465.3 (3607–5.183) μm total length. Body gradually attenuated forward (Fig. 1A); anterior end with three small lips and cephalic papillae (Fig. 1B). Vulva placed one-fifth of body length from posterior end, opening into middle part of ovijector (Fig. 1C). Cuticle with annular striations and without longitudinal ridges (Fig. 1D). Females with conical pointed posterior end, males with trilobate bursal membrane with dorsal lobe reduced and dorsal ray branching at the distal end; spicules slightly equal, short (Fig. 1E): right spicule 134 ± 4.83 (123.2–140) μm, left spicule 131.7 ± 5.32 (123.5–141.3) μm twisted, with triangular structure at the end. Gubernaculum 66.5 ± 5.78 (56–73) μm in length. Eggs (n = 10) oval 86 ± 2.89 (81.6–89.3) μm × 47 ± 1.19 (45.7–47.7) μm (Fig. 1 F).

Concerning the flotation results of lemur's feces, one pool out of two sampled in June 2021, both pools collected in July 2022 and five out of 10 single fecal specimens sampled in November 2022 were positive for strongyle eggs. The feces subjected to Mc Master technique showed <20 to 200 epg (eggs per gram). Both the goat samples tested positive for trichostrongylid eggs together with *Eimeria* spp. oocysts. After the coprocoltures followed by Baermann technique, a huge number of larvae were detected, most of them free living nematodes; after the MAAF method only two third stage larvae from lemurs and one from goats were collected under the dissecting microscope and used for molecular identification.

**Fig. 2:**
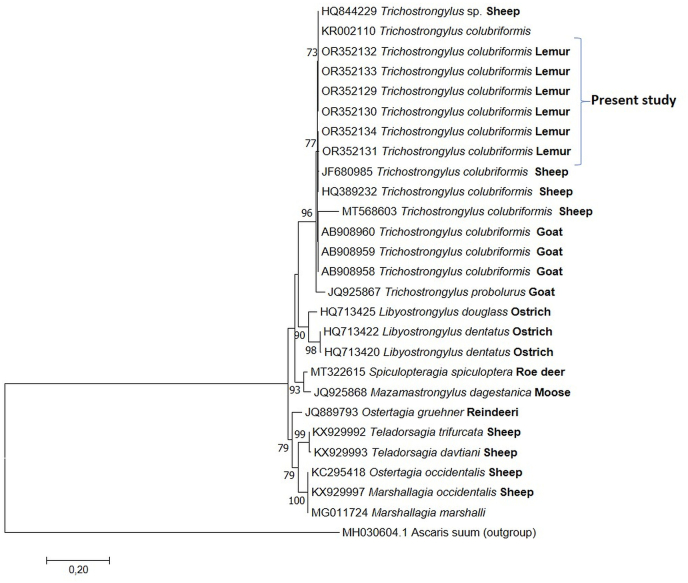
The evolutionary history was inferred by using the Maximum Likelihood method based on the Tamura 3-parameter model. The tree is drawn to scale, with branch lengths measured in the number of substitutions per site. The analysis involved 27 nucleotide sequences. There were a total of 684 positions in the final dataset.

Regarding the molecular analysis, only three out of 20 adults subjected to PCR showed amplifications, while one specimen of strongylid eggs and two third-stage larvae (from coprocolture) showed good amplifications. All the sequences were identical to each other and the comparisons by BLAST gave 99.7% identity with *T. colubriformis*, confirming the morphological results obtained for adult stages. The ML tree from *T. colubriformis* showed that our specimens form a well-supported cluster with the same species retrieved from GenBank, clearly separated from the other strongylid (Fig. 2). The sequence from goat nematodes eggs showed a poor quality pherogram probably due to a mixed infection. Finally, the third stage larva from goats showed 97% identity with *Oesophagostomum venulosum* (Table 1).

## 4 Discussion

We analyzed the entire intestine of a captive ring-tailed lemur which had died naturally detecting the presence of adults of *T*. *colubriformis*, a nematode never reported before in this host. This worm is a common intestinal nematode of ruminants with a worldwide distribution, described also in camelids and primates (Travassos, 1921). The presence of unidentified Trichostrogilids egg has been already reported in *L. catta* (Loudon and Sauther, 2013) but no previous description of *T. colubriformis* was made in this host. Moreover, this species has zoonotic importance, since human infections have been widely reported in several countries, and are commonly linked to the ingestion of unwashed vegetables contaminated with animal feces (Lattès et al., 2011; Sato et al., 2011; Souza et al., 2013; Hidalgo et al., 2020).

The finding of adults in the gut and the results of the faecal analysis in the lemur colony showed that infection with *T. colubriformis* was not an isolated event: in fact, the parasite has been detected in all the sampling activities throughout three years, pointing out how *T. colubriformis* performs its own life cycle within this population of captive lemurs.

Typically, small ruminants are specific hosts of *T. colubriformis*, and in the zoo under study a permanent population of free-roaming goats is present in the pedestrian areas. The visitors' or operators' shoes can therefore easily serve as a vehicle for the contamination of lemur's area. The high degree of terrestriality observed among the ring-tailed lemur population increases the probability of acquiring soil-transmitted parasites. This aspect is very important and should be taken into consideration in zoos, due to the possibility of interspecies transmission as already reported in these settings.

The coprological analysis carried out from the goat feces showed the presence of Trichostrongylidae/Chabertidae eggs showing very similar morphological features among the gastrointestinal nematodes, and cannot be identified properly at species level (Deplazes et al., 2016). Moreover, the molecular analysis performed on the eggs showed a mixed pherogram, probably for the presence of a mixed infection. The third stage larvae collected after the coprocolture, amplified and sequenced was identified as *Oesophagostomum venulosum* (97% similarity), probably as the most abundant species infecting the free-ranging goats.

On the contrary, the molecular analyses performed on the eggs and the third stage larvae obtained from the coprocolture of lemur's feces gave a very clean pherograms and the sequences obtained showed 100–99.7% similarity with *T. colubriformis* as the prevalent species infecting this host.

Walkthrough enclosures are places in which visitors, keepers, and veterinarians encounter animals, but could favor the spreading of some diseases including the zoonotic ones.

For example, *Giardia duodenalis*, has been frequently described in non-human primate species hosted in zoological gardens and the lack of host specificity could imply a risk of infection among the housed animals or between animals and humans (Berrilli et al., 2011; Fomsgaard et al., 2020; Mravcová et al., 2021).

The findings here reported highlight the importance of testing zoo animals by correct sampling and specific identification of parasites, to evaluate their pathogenic role in the housed animals and the zoonotic risk linked to their presence.

Our study reported for the first time *T. colubriformis* infection in ring-tailed lemur, providing evidence of *L. catta* as suitable host for the maintenance of the parasite, with the establishment of a persistent and localized cycle within the hosted population.

Finally, we stress how the access to free-ranging enclosures in combination with the presence of different animal species could facilitate interspecies transmission of different parasites, including zoonotic ones, and this critical aspect must be considered carefully in the management of zoos and in the organization of access to the various areas.

## 5 Conclusions

In conclusion, our study presents a novel observation of *T*. *colubriformis* infection in captive ring-tailed lemurs. This nematode, commonly found in ruminants and with zoonotic significance, has been identified as a persistent and localized infection within the lemur population. The detection of *T. colubriformis* in the lemurs' gastrointestinal tract, along with the parasite's identification through molecular analyses form feces, underscores the importance of accurate parasitological assessments in zoological settings. Furthermore, the potential for interspecies transmission of parasites, including zoonotic agents, within walkthrough enclosures need careful consideration for disease management strategies. By shedding light on this dynamic, our study contributes to the broader understanding of parasite-host interactions in captive environments and the associated zoonotic risks.

## Authors' contributions

Filippo Maria Dini, Monica Caffara and Roberta Galuppi wrote the main manuscript text, Filippo Maria Dini and Roberta Galuppi performed sampling, fecal analysis and light microscopy observation, Filippo Maria Dini and Monica Caffara carried out the molecular analyses. Perla Tedesco provided the technical support for SEM and prepared all the figures. Matteo Galliani, Chiara Cotignoli and Michele Capasso allowed and supported the sampling. Filippo Maria Dini, Monica Caffara, Roberta Galuppi and Perla Tedesco revised the manuscript. All authors reviewed the manuscript and approved the final manuscript.

## Declaration of competing interest

The authors declare that they have no known competing financial interests or personal relationships that could have appeared to influence the work reported in this paper.
